# Supplementary material for: Safeguarding genome integrity during gene-editing therapy in a mouse model of age-related macular degeneration
Source: Nat Commun. 2022 Dec 22;13:7867. doi: 10.1038/s41467-022-35640-4 (PMC9780342; doi:10.1038/s41467-022-35640-4)
Supplement: Supplementary file 1 — Supplementary Information [file 41467_2022_35640_MOESM1_ESM.pdf]

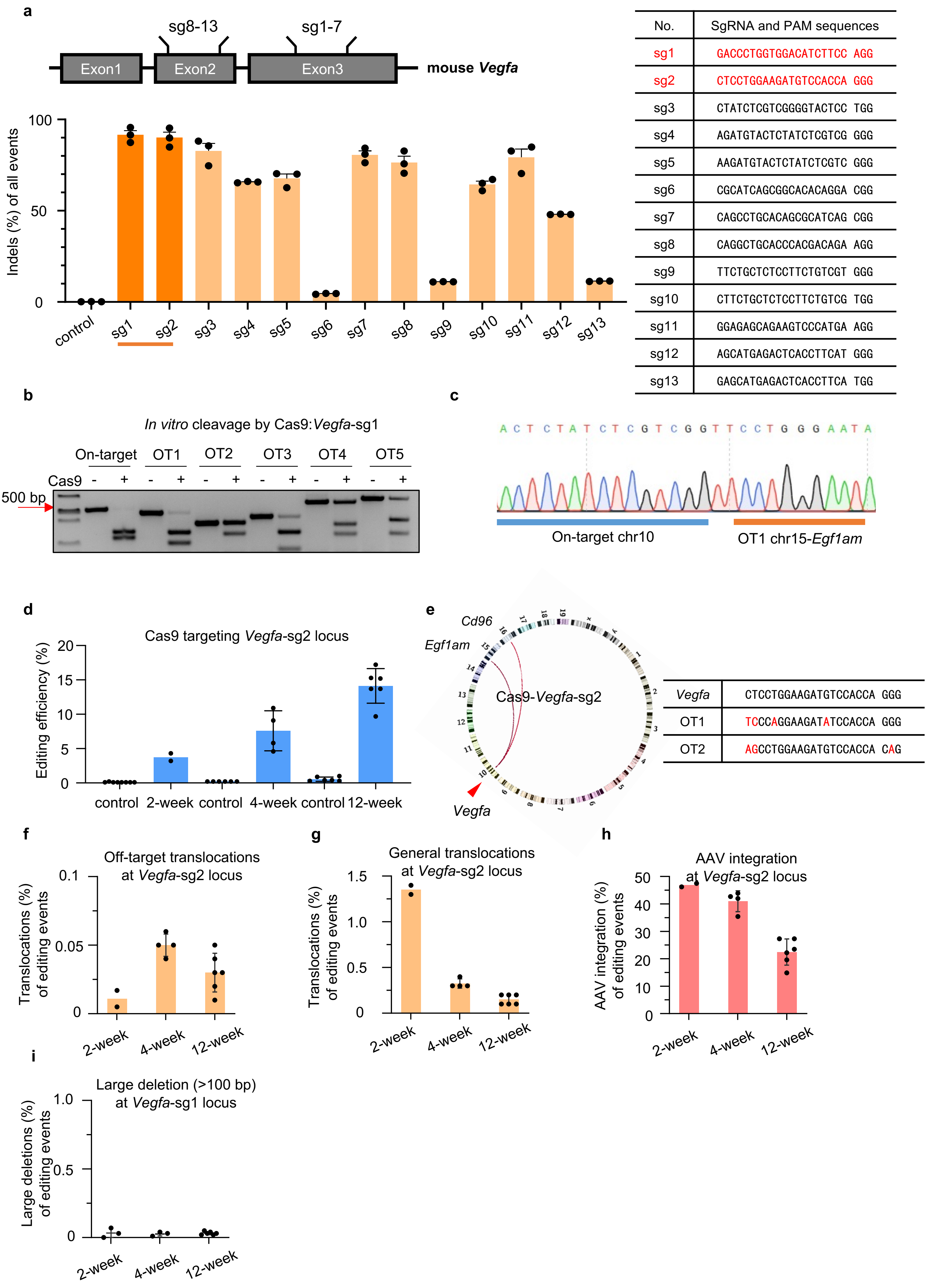

No.	SgRNA and PAM sequences
sg1	GACCCTGGTGGACATCTTCC AGG
sg2	CTCCTGGAAGATGTCCACCA GGG
sg3	CTATCTCGTCGGGGTACTCC TGG
sg4	AGATGTACTCTATCTCGTCG GGG
sg5	AAGATGTACTCTATCTCGTC GGG
sg6	CGCATCAGCGGCACACAGGA CGG
sg7	CAGCCTGCACAGCGCATCAG CGG
sg8	CAGGCTGCACCCACGACAGA AGG
sg9	TTCTGCTCTCCTTCTGTCGT GGG
sg10	CTTCTGCTCTCCTTCTGTCG TGG
sg11	GGAGAGCAGAAGTCCCATGA AGG
sg12	AGCATGAGACTCACCTTCAT GGG
sg13	GAGCATGAGACTCACCTTCA TGG

Vegfa	CTCCTGGAAGATGTCCACCA GGG
OT1	TCCCAGGAAGATATCCACCA GGG
OT2	AGCCTGGAAGATGTCCACCA CAG

Supplementary Figure 1 Chromatin structural variations detected in the mouse caused by Cas9. **a.** Percentage of indels for indicated sgRNAs targeting the exons 2 and 3of *Vegfa* detected by targeting sequencing in N2a cells. Mean ± SD from three biological replicates. SgRNA1 and SgRNA2 were selected for the following study. SgRNA sequences are listed on the right. **b.** Representative *in vitro* cleavage for off-target of *Vegfa*-sg1 by Cas9:*Vegfa*-sg1. Three replicates were performed. **c.** DNA sequence for chromosomal translocations at *Vegfa*-sg1 detected by Sanger sequencing. **d**. Editing efficiency for *Vegfa*-sg2 in mouse eye at 2-, 4- and 12-weeks post-transfection detected by PEM-seq. Error bars, mean ± SD (For 2-, 4-, and 12-weeks post-PBS injection mice, n=8, 6, 6, respectively; for 2-, 4-, and 12-weeks post-AAV injection mice, n=2, 4, 6, respectively). **e**. Circos plot indicating translocations among on-target and off-targets of *Vegfa*-sg2 in mouse eye cells detected by PEM-seq. DNA sequences for on-target and off-targets of *Vegfa*-sg2 are shown in the table. Mismatches of DNA sequences between on-target and off-targets are in red. **f** and **g**. Percentages for off-target translocations (**f**) and general translocations (**g**) in mouse eye cloned from *mVegfa*-sg2 at 2-, 4- and 12-weeks post-transfection detected by PEM-seq. Error bars, mean ± SD (For 2-, 4-, and 12-weeks post-AAV injection mice, n=2, 4, 6, respectively). **h.** Percentages of AAV integrations at *Vegfa*-sg2 normalized to editing events at 2-, 4- and 12-weeks post-transfection detected by PEM-seq. Error bars, mean ± SD (For 2-, 4-, and 12-weeks post-AAV injection mice, n=2, 4, 6, respectively). **i**. Percentages of large deletions at *Vegfa*-sg1 at 2-, 4- and 12-weeks post-transfection detected by PEM-seq. Error bars, mean ± SD (For 2-, 4-, and 12-weeks post-AAV injection mice, n=2, 4, 6, respectively).

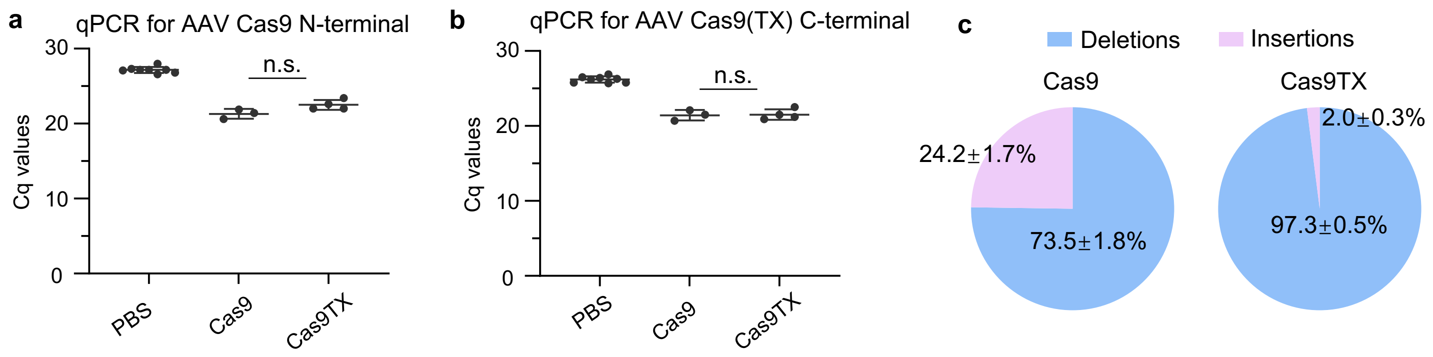

Supplementary Figure 2 Cas9TX slightly outperforms Cas9 in editing efficiencies.
a and **b**. Cq values for Cas9 N-terminal (**a**) and Cas9(TX) C-terminal (**b**) detected by RT-PCR for the indicated groups. Mean ± SD from 8 samples for PBS group, 3 samples for Cas9-treated group, and 4 samples for Cas9TX-treated group. Two-tailed t-test, n.s. means no significance. **c**. Percentages for deletions and insertions in Cas9- and Cas9TX-treated mouse models of AMD at *Vegfa*-sg1.

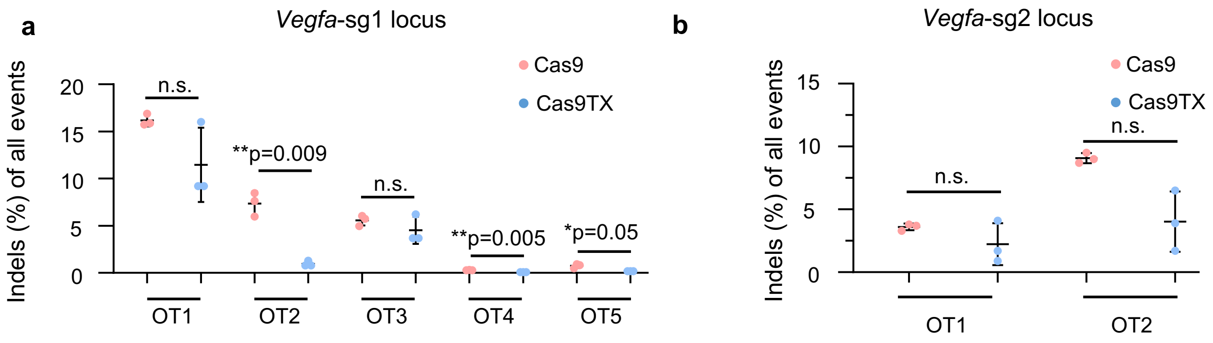

Supplementary Figure 3 Cas9TX induces similar off-target cleavage as Cas9.
a and **b**. Percentage of indels at *Vegfa*-sg1 (a) and *Vegfa*-sg2 (b) loci in mouse eyes edited by Cas9 and Cas9TX detected by target sequencing. Mean ± SD from 3 samples for both *Vegfa*-sg1 and *Vegfa*-sg2 locus. Two-tailed t-test, $*p<0.05$, $**p<0.01$, n.s. means no significance.

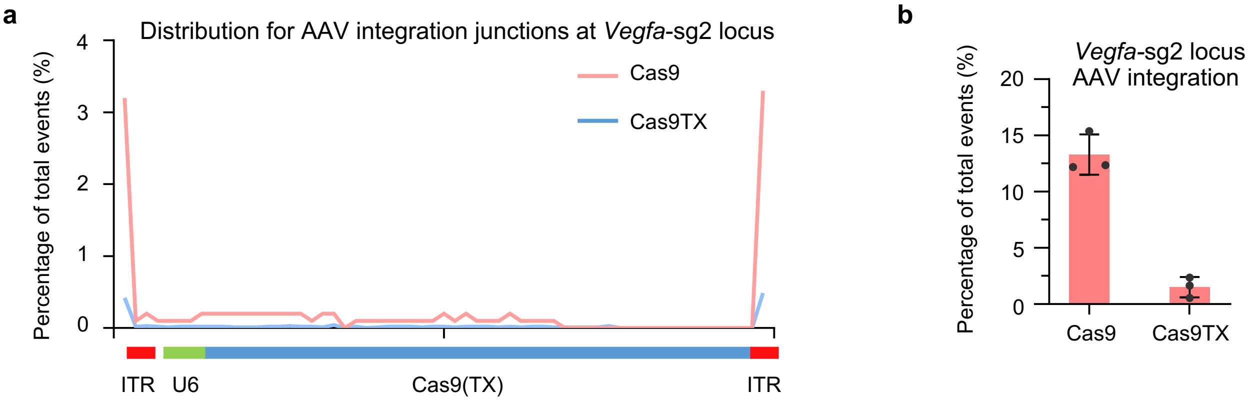

Supplementary Figure 4 Cas9TX greatly reduces AAV integration at the target site.
a. Distribution for AAV integration junctions at the AAV vectors. **b**. Frequency for AAV integrations at *Vegfa*-sg2 for the indicated groups in mouse models of AMD detected by PEM-seq. Mean ± SD from three biological replicates.
