# Supplementary material for: Safeguarding genome integrity during gene-editing therapy in a mouse model of age-related macular degeneration
Source: Nat Commun. 2022 Dec 22;13:7867. doi: 10.1038/s41467-022-35640-4 (PMC9780342; doi:10.1038/s41467-022-35640-4)
Supplement: Supplementary file 3 — Reporting Summary [file 41467_2022_35640_MOESM3_ESM.pdf]

Corresponding author(s): Jiazhi Hu
Last updated by author(s): Nov 23, 2022

# Reporting Summary

Nature Portfolio wishes to improve the reproducibility of the work that we publish. This form provides structure for consistency and transparency in reporting. For further information on Nature Portfolio policies, see our Editorial Policies and the Editorial Policy Checklist.

## Statistics

For all statistical analyses, confirm that the following items are present in the figure legend, table legend, main text, or Methods section.

| n/a | Confirmed | |
|---|---|---|
| ☐ | ☒ | The exact sample size (*n*) for each experimental group/condition, given as a discrete number and unit of measurement |
| ☐ | ☒ | A statement on whether measurements were taken from distinct samples or whether the same sample was measured repeatedly |
| ☐ | ☒ | The statistical test(s) used AND whether they are one- or two-sided<br>*Only common tests should be described solely by name; describe more complex techniques in the Methods section.* |
| ☐ | ☒ | A description of all covariates tested |
| ☐ | ☒ | A description of any assumptions or corrections, such as tests of normality and adjustment for multiple comparisons |
| ☐ | ☒ | A full description of the statistical parameters including central tendency (e.g. means) or other basic estimates (e.g. regression coefficient) AND variation (e.g. standard deviation) or associated estimates of uncertainty (e.g. confidence intervals) |
| ☐ | ☒ | For null hypothesis testing, the test statistic (e.g. *F*, *t*, *r*) with confidence intervals, effect sizes, degrees of freedom and *P* value noted<br>*Give P values as exact values whenever suitable.* |
| ☒ | ☐ | For Bayesian analysis, information on the choice of priors and Markov chain Monte Carlo settings |
| ☒ | ☐ | For hierarchical and complex designs, identification of the appropriate level for tests and full reporting of outcomes |
| ☒ | ☐ | Estimates of effect sizes (e.g. Cohen's *d*, Pearson's *r*), indicating how they were calculated |

*Our web collection on statistics for biologists contains articles on many of the points above.*

## Software and code

Policy information about availability of computer code

| | |
|---|---|
| Data collection | Next-generation data was collected and demutiplexed by Illumina Hiseq X-Ten. |
| Data analysis | All custom code used for PEM-seq analysis are available: https://github.com/liumz93/PEM-Q<br>Percentage of indels for off-target for Cas9 and Cas9TX was analyzed by CRISPResso (version 1.0.8).<br>DNA electrophoresis was shown by Image J (version 1.51 J8). |

For manuscripts utilizing custom algorithms or software that are central to the research but not yet described in published literature, software must be made available to editors and reviewers. We strongly encourage code deposition in a community repository (e.g. GitHub). See the Nature Portfolio guidelines for submitting code & software for further information.

## Data

Policy information about availability of data

All manuscripts must include a data availability statement. This statement should provide the following information, where applicable:

- Accession codes, unique identifiers, or web links for publicly available datasets
- A description of any restrictions on data availability
- For clinical datasets or third party data, please ensure that the statement adheres to our policy

Original PEM-seq data for mouse eye were deposited into the National Omics Data Encyclopedia (NODE) (OEP000911). Source data are provided with this paper.

## Human research participants

Policy information about studies involving human research participants and Sex and Gender in Research.

| | |
|---|---|
| Reporting on sex and gender | n/a |
| Population characteristics | n/a |
| Recruitment | n/a |
| Ethics oversight | n/a |

Note that full information on the approval of the study protocol must also be provided in the manuscript.

# Field-specific reporting

Please select the one below that is the best fit for your research. If you are not sure, read the appropriate sections before making your selection.

☒ Life sciences ☐ Behavioural & social sciences ☐ Ecological, evolutionary & environmental sciences

For a reference copy of the document with all sections, see nature.com/documents/nr-reporting-summary-flat.pdf

# Life sciences study design

All studies must disclose on these points even when the disclosure is negative.

| | |
|---|---|
| Sample size | No sample size calculation was performed. Based on previous studies, at least three replicates were performed for PEM-seq analysis in mouse and N2a cell lines as previous done (Yin et al., 2022, Nature Communications; PMID: 35260581). And at least 8 mice for each group were analysis for VEGFA detection and CNV. |
| Data exclusions | Samples with low editing efficiency (<1%) were removed, for AAV injection for retina cannot be precisely controlled by each cohort. |
| Replication | All replication attempts were successful and two different sgRNAs were performed in mouse to ensure the robustness. Moreover, our findings for Cas9TX are consistent with previous results in primary T cells. |
| Randomization | Randomization is not relevant to the studies. Most samples are prepared from cell lines or animals, sizes are small and the result are consistent. |
| Blinding | No blinding was performed due to the obvious different effect of Cas9TX in comparison with Cas9. Moreover, PEM-seq analysis can self confirm which nuclease edited the sample. |

# Reporting for specific materials, systems and methods

We require information from authors about some types of materials, experimental systems and methods used in many studies. Here, indicate whether each material, system or method listed is relevant to your study. If you are not sure if a list item applies to your research, read the appropriate section before selecting a response.

## Materials & experimental systems

| n/a | Involved in the study | |
|---|---|---|
| ☒ | ☐ | Antibodies |
| ☐ | ☒ | Eukaryotic cell lines |
| ☒ | ☐ | Palaeontology and archaeology |
| ☐ | ☒ | Animals and other organisms |
| ☒ | ☐ | Clinical data |
| ☒ | ☐ | Dual use research of concern |

## Methods

| n/a | Involved in the study | |
|---|---|---|
| ☒ | ☐ | ChIP-seq |
| ☒ | ☐ | Flow cytometry |
| ☒ | ☐ | MRI-based neuroimaging |

## Eukaryotic cell lines

Policy information about cell lines and Sex and Gender in Research

| | |
|---|---|
| Cell line source(s) | Neuro-2A cell line was from National Collection of Authenticated Cell Cultures, Chinese Academy of Science. HEK293T was a |

| | |
|---|---|
| Cell line source(s) | gift from Dr. Frederick Alt Lab (Harvard Medical School), which was from ATCC. |
| Authentication | Cell lines were confirmed by STR (short tandem repeat). |
| Mycoplasma contamination | Mycoplasma contamination was negative. |
| Commonly misidentified lines (See ICLAC register) | No |

## Animals and other research organisms

Policy information about studies involving animals; ARRIVE guidelines recommended for reporting animal research, and Sex and Gender in Research

| | |
|---|---|
| Laboratory animals | The care and use of animals were reviewed and approved by the Institutional Animal Care and Use Committee (IACUC) of HuiGene Therapeutics Co., Ltd. |
| Wild animals | The study did not involve wild animals. |
| Reporting on sex | Adult (6-8 week old) male and female SPF C57BL/6J mice (half and half) were used in the study. |
| Field-collected samples | Animals were housed socially in standard cages with a feeding box and a water bottle. The environment of the laboratory animal facilities and cages used were in accordance with China National Standard: GB14925-2010 – Laboratory Animals – Requirements of Environment and Housing Facilities (2010-12-23). The rooms were controlled and monitored for humidity (targeted range 40% - 70%, actual range 20.6% - 77.5%) and temperature (targeted range 18℃ - 26℃, actual range 17.6℃ - 25.1℃) with 10 to 20 air changes/hour. The room was on a 12-hour light/dark cycle except when interruptions were required by study activities. The rooms in which the animals were housed have been documented in the facility records. |
| Ethics oversight | All animal experiments were conducted in compliance with the Guide for the Laboratory Animal Administration Regulation (2017) issued by the National Science and Technology Committee (China) and the laboratory animal administration regulations (Shanghai, Jiangsu). The care and use of animals were reviewed and approved by the Institutional Animal Care and Use Committee (IACUC) of HuiGene Therapeutics Co., Ltd. |

Note that full information on the approval of the study protocol must also be provided in the manuscript.
